# 3D printed Ti-5Cu alloy accelerates osteogenic differentiation of MC3T3-E1 cells by stimulating the M2 phenotype polarization of macrophages

**DOI:** 10.3389/fimmu.2022.1001526

**Published:** 2022-10-07

**Authors:** Xin Zhao, Xing Zhou, Hui Sun, Huixin Shi, Yiping Song, Qiang Wang, Guangping Zhang, Dake Xu

**Affiliations:** ^1^ School and Hospital of Stomatology, China Medical University, Liaoning Provincial Key Laboratory of Oral Diseases, Shenyang, China; ^2^ Shenyang National Laboratory for Materials Science, Northeastern University, Shenyang, China; ^3^ Electrobiomaterials Institute, Key Laboratory for Anisotropy and Texture of Materials (Ministry of Education), Northeastern University, Shenyang, China; ^4^ Department of Plastic Surgery, The First Hospital of China Medical University, Shenyang, China; ^5^ Department of Stomatology, Shengjing Hospital of China Medical University, Shenyang, China

**Keywords:** 3D printing technology, Ti-5Cu alloy, macrophage polarization, oncostatin M, osteogenic differentiation, bone immunomodulation

## Abstract

Ti-5Cu alloy has been proved to have excellent mechanical properties and cell compatibility and has certain antibacterial properties due to the addition of Cu. However, there are few studies on the effects of Ti-5Cu alloy on macrophage polarization and immune-related bone formation. In this study, we prepared Ti-5Cu alloy by three-dimensional printing technology and found that Ti-5Cu alloy presented a much smoother surface compared with Ti. In addition, the CCK-8 results indicated the Ti-5Cu alloy had no cytotoxicity to RAW264.7 cells by co-culture. The results of inductively coupled plasma mass spectrometry showed that the concentration of Cu^2+^ was 0.133 mg/L after 7 days of co-culture, and the CCK-8 results proved that Cu^2+^ had no cytotoxicity to RAW264.7 at this concentration. Then, we studied the effects of Ti-5Cu alloy on macrophage polarization; it was shown that the Ti-5Cu alloy is more prone to modulate the RAW264.7 polarization towards the M2 phenotype and the conditioned medium derived from Ti-5Cu alloy significantly promoted the proliferation and osteogenic differentiation of MC3T3-E1 cells. However, when the expression of Oncostatin M (OSM) gene of RAW264.7 was knocked down, the osteogenic differentiation of MC3T3-E1 cells was decreased. This suggests that the OSM secreted by RAW264.7 co-cultured with Ti-5Cu alloy could accelerate the osteogenic differentiation of MC3T3-E1 cells by acting on OSMR/gp130 receptors.

## 1 Introduction

Maxillofacial bone defect is a common disease in oral surgery. It can lead to abnormal appearance or dysfunction in the maxillofacial region, which greatly affects the quality of life. At present, there are several treatments for the disease, like autogenous bone grafts, allogeneic bone grafts, and artificial bone substitutes. However, autogenous bone grafts may cause severe trauma, allografts or xenografts can lead to immune rejection or viral infection, and artificial bone substitutes cannot accurately restore the normal anatomical morphology of maxillofacial bone tissue (1).

In recent years, the development of three-dimensional (3D) printing technology has made it possible to personalize the repair of patients with maxillofacial bone defects. It can accurately print bone substitutes that match the bone defect sites. After systematic evaluation, bone substitutes can be implanted into the human body (2). However, after implanted into the body, most bone implants will inevitably stimulate the immune response. We hope that the immune response induced by bone implants and the created osteo-immune microenvironment can stimulate osteogenesis (3).

It is widely known that macrophages are the key cells in response to the immune environment. They can polarize into different phenotypes according to different stimulation in the local microenvironment. Macrophages are typically divided into two phenotypes. For example, intracellular pathogens or Lipopolysaccharide (LPS) and Interferon γ (IFN-γ) *in vivo* can induce macrophages into M1 type, called “classically activated macrophages”, and some parasites and immune complexes or Interleukin 4 (IL-4) can induce macrophages into M2 type, called “alternatively activated macrophages”. Among them, M1 type has highly expression of CD86 and Inductible Nitric Oxide Synthase (iNOS) and secretes inflammatory cytokines, such as Tumor Necrosis Factor α (TNF-α) and IL-1β, and some other chemokines. It is mainly involved in the pro-inflammatory reaction and causes tissue damage. Conversely, CD206, Arg-1, and some other anti-inflammatory cytokines like IL-10 are highly expressed in M2-type macrophages that play an important role in anti-inflammatory reaction and participate in tissue healing (4).

However, the phenotype of macrophage also shows a dynamic change during bone healing. Bone healing can be divided into three stages: inflammation, repairing, and remodeling. In the inflammatory stage, macrophages are mainly polarized into M1 type and then polarized into M2 type generally after 72 h (5). In view of the functional plasticity of macrophages, scholars hope to regulate the polarization types of macrophages by modifying biomaterials. Through changing the surface roughness, preparing porous materials or adding various nutritional elements enables immunomodulatory properties of biomaterials, so as to promote tissue regeneration (1). The surface roughness and hydrophilicity of biomaterials affect the polarization of macrophages. Li et al. (6) investigated the effects of mineralized collagen (MC) with different surface roughness on the macrophage polarization, and they found that MC with higher surface roughness tended to polarize macrophages to the M1 phenotype, whereas macrophages grown on smoother surfaces exhibited the M2 phenotype. Meanwhile, Hamlet et al. (7) compared the effect of hydrophilic titanium alloy surface on macrophage function and found that the more hydrophilic titanium alloy surface could reduce the expression of pro-inflammatory factors and promote the expression of anti-inflammatory factors in macrophages. The different surface roughness and hydrophilicity of the biomaterials surface can affect the amount and structure of serum proteins adsorbed on the its surface and then affect the immune response of macrophages (7). Activation of macrophage surface receptors, such as integrins, and Wnt signaling pathway contribute to the macrophage polarization regulated by biomaterials (8).

As we know, Cu is one of the essential trace elements in the human body; it plays an important role in maintaining physiological stability and cellular immunity (9). Infection after implantation in maxillofacial region and peri-implantitis caused by microorganisms often lead to implant failure (10). As an antibacterial element, Cu has been added to pure titanium to endow the implants with certain antibacterial properties (11). In one of our previous works, the effects of 3, 5, and 7 wt% Cu addition in titanium alloy on the osteogenic properties of MC3T3-E1 cells and its antibacterial properties were compared under the same treatment condition. The results showed that the antibacterial properties and osteogenic properties of Ti-Cu alloy improved with the increase of Cu content. However, the cytocompatibility of Ti-7Cu was significantly inferior to that of Ti-5Cu and Ti-3Cu, which showed that the cell proliferation rate was decreased and the early apoptosis rate was increased (12). Liu et al. (13) showed that only when the content of Cu in Ti-Cu alloy is greater than 5 wt%, the alloy can exhibit certain antibacterial properties. Another *in vitro* and *in vivo* study illustrated that Ti-5Cu alloy has excellent corrosion resistance similar to Ti, which can inhibit bone resorption caused by bacterial infection and enhance bone formation; in addition, their previous study also optimized the Cu content of Ti-Cu alloy and found that 5 wt% Cu had better biocompatibility and antibacterial properties while maintaining the mechanical properties of Ti (14). These studies indicated that Ti-5Cu alloy can be used in maxillofacial defect repair and oral implants due to its good cytocompatibility, antibacterial properties, and excellent mechanical properties, but whether the 3D printed Ti-5Cu alloy that contains 5 wt% Cu will regulate the polarization types of macrophages is still unknown.

Macrophages are functionally heterogeneous cells, which can secrete a variety of cytokines after co-culture with different materials. Studies have shown that cytokines secreted by macrophages also have different effects on bone formation (15). A study by Champagne et al. (16) revealed that Bone Morphogenetic Protein 2 (BMP2) secreted by macrophages could increase alkaline phosphatase (ALP) expression in hMSCs, thus promoting the wound healing. Moreover, another study discovered that Transforming Growth Factor β (TGF-β) secreted by polarized macrophages is also a key factor to promote the osteogenic differentiation of Bone Marrow Mesenchymal Stem Cells (BMSCs) (17). Otherwise, Oncostatin M (OSM) is one of the newly discovered bone reconstruction regulatory proteins (18). It is mainly secreted by macrophages, T cells, and dendritic cells and has a variety of biological activities, such as promoting bone formation, stimulating hematopoiesis, and promoting cell proliferation (19, 20). OSMR/gp130 and LIFR/gp130 are the two main receptors of OSM; OSMR, as one of the specific receptors, is mainly distributed on the surface of bone marrow stem cells and osteoblasts (21). Studies have shown that OSM can activate Jak/Stat, Erk1/2, Yap1, Notch, and PKCδ signal pathways through OSMR/gp130 receptors (22–24).

Therefore, in this study, we added 5 wt% Cu into pure Ti through 3D printing technology to investigate the effects of Cu addition on macrophage polarization and the osteogenesis mechanism of cytokines secreted by macrophages on MC3T3-E1 cells. We hope that the addition of 5 wt% Cu can provide Ti-5Cu alloy with a better immunomodulation microenvironment, thus promoting bone regeneration.

## 2 Materials and methods

### 2.1 Materials preparation

The Ti and Ti-5Cu alloy were prepared by selective laser melting technology; the fabrication details were consistent with our previous work (25). Briefly, commercial Ti powder and Cu powder were fabricated into Ti-5Cu alloy; the Ti alloy was fabricated as the same processing without using Cu powder. The samples with a size of 10 × 10 × 1.5 mm were used in this experiment. Before the experiment, all the samples were polished to 2,000 grits with SiC sandpaper, then ultrasonically cleaned with deionized water and anhydrous ethanol for 10 min each, dried at room temperature, and finally sterilized at 126° C for 30 min for subsequent experiments.

### 2.2 Surface characterization

The surface morphology and element components of samples were characterized by scanning electron microscope (SEM) equipped with Energy Dispersive Spectrometer (EDS) (Zeiss, Germany). The x-ray diffractometer (XRD; Philips PW1700, The Netherlands) was used to analyze the phase composition of samples at a scanning speed of 5°/min ranging from 20° to 90° using CuKα radiation. The 3D surface roughness of Ti and Ti-5Cu alloy was detected by the laser scanning confocal 3D microscope (Olympus OLS4000, Japan). The contact angles of samples were measured by a contact angle instrument (TBU 95, Data Physics, Germany), 2 μl of distilled water was dropped onto the surface of samples, and the contact angles of three different areas on the surface of samples were measured with SCA20 software. For surface roughness and contact angles tests, three samples were taken, and four points were randomly selected for measurements. For Cu^2+^ release test, six pieces of Ti and Ti-5Cu alloys were immersed in the Dulbecco's Modification of Eagle’s Medium (DMEM) complete culture medium in six-well culture plates at 37° C. Each group had three parallel wells. The culture medium from three parallel wells of each group was collected together at each time point. The inductively coupled plasma mass spectrometry (ICP-MS; USA) was used to measure the concentration of Cu^2+^ at 1, 3, and 7 days.

### 2.3 Cell culture

The RAW264.7 cells were cultured in DMEM complete culture medium (Gibco, USA) containing 10% Fatal Bovine Serun (FBS) (Clark, USA) and 1% penicillin/streptomycin (Gibco, USA). The MC3T3-E1 cells were cultured in Alpha Modification of Minimum Essential Medium (α-MEM) complete culture medium (Meilun, China) containing 10% FBS (Clark, USA) and 1% penicillin/streptomycin (Gibco, USA). All the cells were cultured in a cell incubator containing 5% CO_2_ at 37° C with constant temperature and humidity. For the α-MEM osteogenic medium, 10 mM β-glycerophosphate disodium, ascorbic acid (50 μg/ml), and 100 nM dexamethasone were added into the α-MEM complete medium. The cells were passaged when they became 80%–90% confluent. The RAW264.7 cells and MC3T3-E1 cells used in this experiment were provided by the Central Laboratory of School and Hospital of Stomatology affiliated to China Medical University.

### 2.4 CCK-8 assay

RAW264.7 cells were seeded on the surface of Ti and Ti-5Cu alloy in 24-well culture plates at a density of 1 × 10^4^ per well. The complete medium without materials was used as the control group. Then, the 24-well culture plates were incubated for 24, 48, and 72 h. At each time point, after discarding the supernatant, 1 ml of DMEM containing 10% CCK-8 reagent (US Everbright, USA) was added to each well. After incubated for 2 h, culture medium was transferred into a new 96-well plate, and four wells were taken from each group for measurements. The optical density (OD) values were measured at 450 nm by a microplate reader (Infinite M200, Tecan, Australia).

The gradient Cu^2+^ culture medium was prepared by CuCl_2_ (Sinopharm, China) with DMEM complete medium; the concentrations are 0.4375, 0.875, 1.75, and 3.5 mg/L. RAW264.7 cells were seeded in 96-well culture plates at a density of 2 × 10^3^ per well, and each group had four parallel wells. After 24 h, the medium was replaced with different concentration of Cu^2+^ culture medium. The DMEM complete medium without Cu^2+^ was used as the control group. Then, the 96-well culture plates were incubated for 24, 48, and 72 h. At each time point, after discarding the supernatant, 100 μl of DMEM containing 10% CCK-8 reagent was added to each well. After incubated for 2 h, the OD values were measured at 450 nm by a microplate reader (Infinite M200, Tecan, Australia).

MC3T3-E1 cells were seeded in 96-well culture plates at a density of 2 × 10^3^ per well, and each group had four parallel wells. After 24 h, the culture medium was replaced with different material extracts or conditioned medium. The 96-well culture plates were incubated for 1, 3, 5, and 7 days. At each time point, after discarding the supernatant, 100 μl of DMEM containing 10% CCK-8 reagent was added to each well. After incubated for 2 h, the OD values were measured at 450 nm by a microplate reader (Infinite M200, Tecan, Australia).

The cell viability was calculated according to the following formula:

Cell viability (%) = (OD_experimental group_ − OD _blank group_)/(OD _control group_ − OD _blank group_) × 100%.

### 2.5 Flow cytometry test

RAW264.7 cells were seeded on the surface of Ti and Ti-5Cu alloy in six-well culture plates at a density of 1 × 10^5^ per well and six pieces of samples in each well. Each group had three parallel wells. The six-well culture plates were incubated for 24 and 72 h. At each time point, cells were collected and washed with Phosphate Buffered Saline (PBS) for three times, stained with Allophycocyanin (APC)-conjugated CD86 (Invitrogen, USA), Phycoerythrin (PE)-conjugated CD206 (Invitrogen, USA), and then incubated for 30 min. After washing with PBS twice, the cells were resuspended with 300 μl of PBS. Finally, the results were acquired using a flow cytometry (BD, USA). The obtained data were analyzed by FlowJo software.

### 2.6 Enzyme-linked immunosorbent assay

RAW264.7 cells were seeded on the surface of Ti and Ti-5Cu alloy in six-well culture plates at a density of 1 × 10^5^ per well and six pieces of samples in each well. Each group had three parallel wells. The supernatant was collected after incubated for 24 and 72 h, respectively, and then centrifuged at 2,000 rpm for 20 min at 4° C; the concentrations of TNF-α, IL-10, and OSM secreted by RAW264.7 cells were quantified by enzyme-linked immunosorbent assay (ELISA) kits (Boster, China) following the manufacturer**’**s instructions.

RAW264.7 cells were seeded in six-well culture plates at a density of 1 × 10^5^ per well; after cultured for 24 h, the supernatants were replaced with the Ti and Ti-5Cu extracts and then cultured for another 72 h; the supernatant was collected to detect the concentration of TNF-α and IL-10 secreted by RAW264.7 cells as the method mentioned above.

### 2.7 RT-qPCR

RAW264.7 cells were seeded on the surface of Ti and Ti-5Cu alloy in six-well culture plates at a density of 1 × 10^5^ per well and six pieces of samples in each well. Each group had three parallel wells. After incubated for 24 and 72 h, respectively, then the total RNA of RAW264.7 cells was extracted by TRIzol reagent (Takara, Japan) according to the manufacturer**’**s instructions. Total RNA (1 μg) was used for synthesizing the cDNA using the PrimerScript RT reagent Kit (Takara, Japan). The gene expression was calculated by the QuantStudio3 (Applied Biosystems, USA) using the TB Green Premix Ex Taq II Kit (Takara, Japan). TNF-α, IL-10, CD86, and CD206 were chosen to evaluate the effects of materials on the polarization of RAW264.7 cells. OSM, TGF-β, and BMP6 were used to evaluate the osteogenic gene expression of RAW264.7 cells. The β-actin was used as a reference gene. Relative quantification was calculated by the comparative 2^−ΔΔCt^ method. The primers for TNF-α, IL-10, CD86, CD206, OSM, TGF-β, BMP6, and β-actin were synthesized by Sangon Biotech (Sangon, China).

RAW264.7 cells were seeded in six-well culture plates at a density of 1 × 10^5^ per well; after cultured for 24 h, the supernatants were replaced with the Ti and Ti-5Cu extracts and then cultured for another 72 h. Total RNA was extracted of each group for PCR amplification as described above to detect the gene expressions of TNF-α, IL-10, and OSM in RAW264.7 cells.

MC3T3-E1 cells were seeded in six-well culture plates at a density of 1 × 10^5^ per well. After 24 h, the culture medium was replaced with different material extracts and conditioned medium. The six-well culture plates were incubated for 7 days, and then, the total RNA was extracted for RT-qPCR detection. The detailed steps are shown as above. ALP, Collagen type I (COL-I), Runt-related transcription factor 2 (Runx2), and Osteocalcin (OCN) were chosen for osteogenesis-related genes; OSMR and gp130 were used to evaluate the OSM signaling pathway–related gene expression; β-actin was used as a reference gene; and they were synthesized by Sangon Biotech (Sangon, China).

MC3T3-E1 cells were seeded in six-well culture plates at a density of 1 × 10^5^ per well. After 24 h, the medium was replaced with OSM-Small interfering RNA (siRNA)–derived conditioned medium and Negative Control (NC)-siRNA–derived conditioned medium. MC3T3-E1 cultured in normal α-MEM osteogenic medium was used as the control group. After incubated for 7 days, the expressions of osteogenesis-related genes (ALP, COL-I, Runx2, and OCN) and OSM signaling pathway–related genes (OSMR, gp130) were analyzed by RT-qPCR method as mentioned above. All of the sequences of primers were listed in **Table 1**.

**Table 1:** Primers used in RT-qPCR.

	Forward (5′-3′)	Reverse (5′-3′)
TNF-α	AGCCCACGTCGTAGCAAAC	GGTGAGGAGCACGTAGTCG
IL-10	GTAGAAGTGATGCCCCAGGC	CACCTTGGTCTTGGAGCTTATT
CD86	CTGTAGGCAGCACGGACTT	CTCCACGGAAACAGCATCTGAG
CD206	CTCTGTTCAGCTATTGGACGC	CGGAATTTCTGGGATTCAGCTTC
OSM	ACTCTTGGAGCCCTATATCCG	AGACTCTGTCCAGTGTGGTG
TGF-β	CTCCCGTGGCTTCTAGTGC	GCCTTAGTTTGGACAGGATCTG
BMP6	TGGCAGGACTGGATCATTGC	ACCAAGGTCTGTACAATGGCG
ALP	CCAACTCTTTTGTGCCAGAGA	GGCTACATTGGTGTTGAGCTTTT
COL-I	CGATGGATTCCCGTTCGAGTA	GTGCTGTAGGTGAAGCGACT
Runx2	TCGGAGAGGTACCAGATGGG	TGAAACTCTTGCCTCGTCCG
OCN	CTGACCTCACAGATCCCAAGC	TGGTCTGATAGCTCGTCACAAG
OSMR	CATCCCGAAGCGAAGTCTTGG	GGCTGGGACAGTCCATTCTAAA
gp130	CCGTGTGGTTACATCTACCCT	CGTGGTTCTGTTGATGACAGTG
β-Actin	GTGCTATGTTGCTCTAGACTTCG	ATGCCACAGGATTCCATACC

### 2.8 Preparation of material extracts and conditioned medium

Six pieces of Ti and Ti-5Cu alloys were immersed in six-well plates containing 2 ml of DMEM complete culture medium; after immersing for 72 h, the extracts were collected for RAW264.7 polarization experiment, and the extracts immersed for 48 h were collected for the osteogenesis experiment of MC3T3-E1 cells.

RAW264.7 cells were co-cultured with Ti and Ti-5Cu alloys for 48 h; the supernatant was collected, centrifuged at 2,000 rpm for 20 min at 4° C, and then filtered by 0.22-μm filter. The collected medium was mixed with α-MEM osteogenic medium at a ratio of 1:1 to prepare the conditioned medium (CM). To eliminate the influence of ions extracted from materials on MC3T3-E1 cells, we prepared the material extracts without RAW264.7 cells. MC3T3-E1 cultured in a α-MEM osteogenic medium was used as the control group.

### 2.9 siRNA transfection

RAW264.7 cells were seeded in six-well culture plates with Ti-5Cu alloy at a density of 1 × 10^5^ per well. The cells were transfected with siRNA when they became 50%–70% confluent. OSM-siRNA and NC-siRNA were purchased from GenePharma (Suzhou, China); the sequences of OSM-siRNA are 5′-CACUCUUGGAGCCCUAUAUTT-3′ (sense) and 5′-AUAUAGGGCUCCAAGAGUGTT-3′ (antisense); the sequences of NC-siRNA are 5′-UUCUCCGAACGUGUCACGUTT-3′ (sense) and 5′-ACGUGACACGUUCGGAGAATT-3′ (antisense). siRNA was transfected into RAW264.7 cells using GP-transfect-Mate (GenePharma, China) according to the manufacturer**’**s instructions. The medium was replaced with DMEM complete medium after 24 h, and then, the supernatant was collected after 48 h to prepare the conditioned medium as mentioned above.

### 2.10 ALP staining

MC3T3-E1 cells were seeded in 24-well culture plates at a density of 2 × 10^4^ per well. After 24 h, the culture medium was replaced with different material extracts and conditioned medium. The 24-well culture plates were incubated for 7 days. Then, the cells were washed once and fixed with 4% paraformaldehyde for 1 h. After washing with PBS twice, the cells were stained with a 5-bromo-4-chloro-3-inodlyl-phosphate/Nitro-Blue-Tetrazolium (BCIP/NBT) ALP color development kit (Beyotime, China) according to the manufacturer’s instructions. Three sites were randomly selected from each well to take photographs by an inverted microscope (Nikon Ts2R, Japan). The ImageJ software was used to quantify the ALP staining results.

### 2.11 Statistical analysis

Results were shown as the mean values ± standard deviation (SD). The data were analyzed by the one-way analysis of variance (ANOVA) followed by the Least Significant Difference (LSD) *t*-test or the Student**’**s *t*-test using SPSS 20 software. Differences were considered as statistically significant when *p* < 0.05.

## 3 Results

### 3.1 Characterization of materials

**Figure 1:**
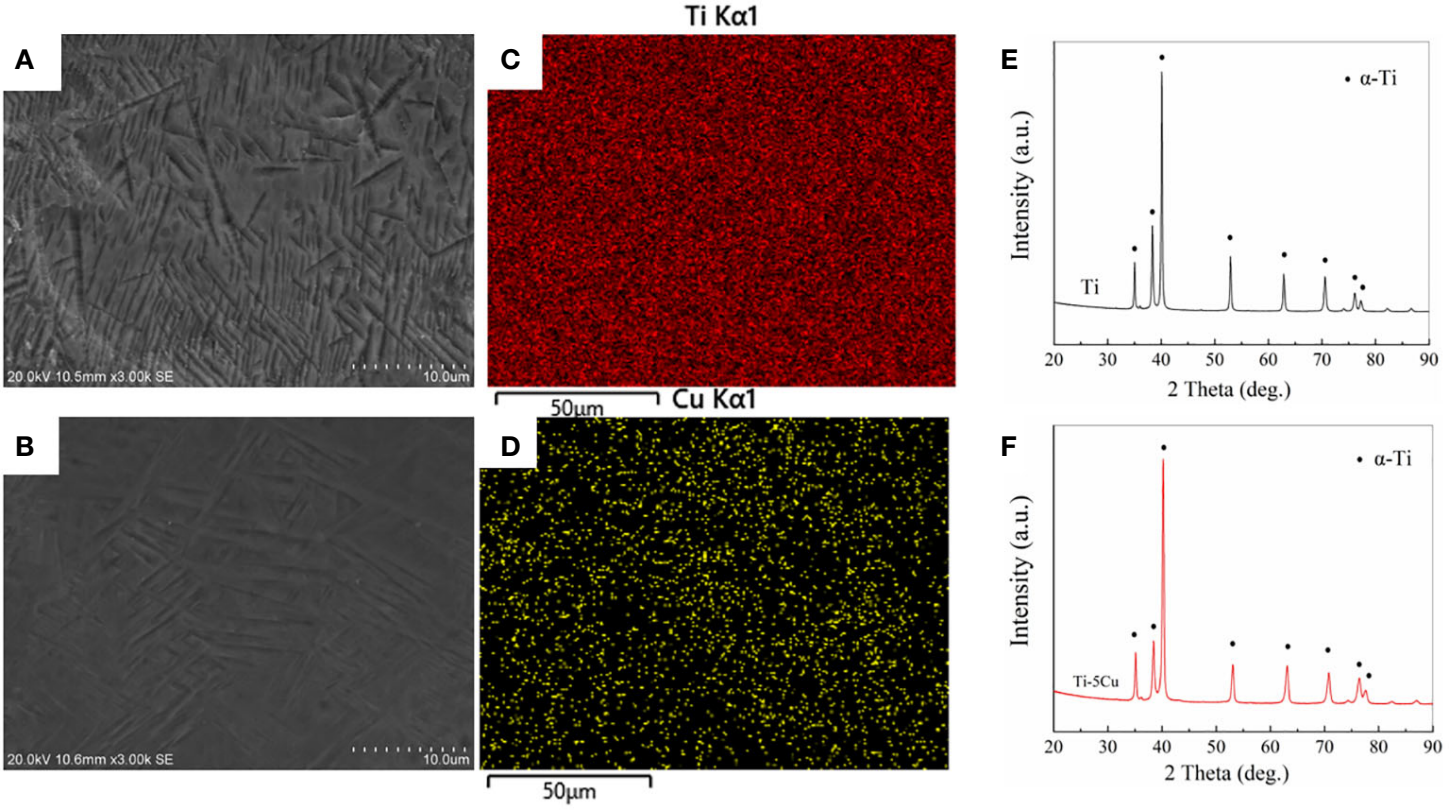
Materials characterization. **(A)** SEM image of Ti; **(B)** SEM image of Ti-5Cu; **(C)** Ti distribution in Ti-5Cu alloy by EDS; **(D)** Cu distribution in Ti-5Cu alloy by EDS; **(E)** XRD patterns of Ti; **(F)** XRD patterns of Ti-5Cu.

The microstructure of the two samples was observed under SEM, as shown in **Figures 1A, B**. The Ti element in both samples presented α phase that had a crossed appearance, and most of the Cu element was solid-dissolved in the α grains after cooling (10). The Ti and Cu elements were uniformly distributed in the matrix, as shown in the EDS results in **Figures 1C, D**. The results of XRD in **Figures 1E, F** showed that the addition of Cu did not change the phase composition of Ti, both of which were mainly α-Ti phase.

### 3.2 Surface roughness and hydrophilicity

**Figure 2:**
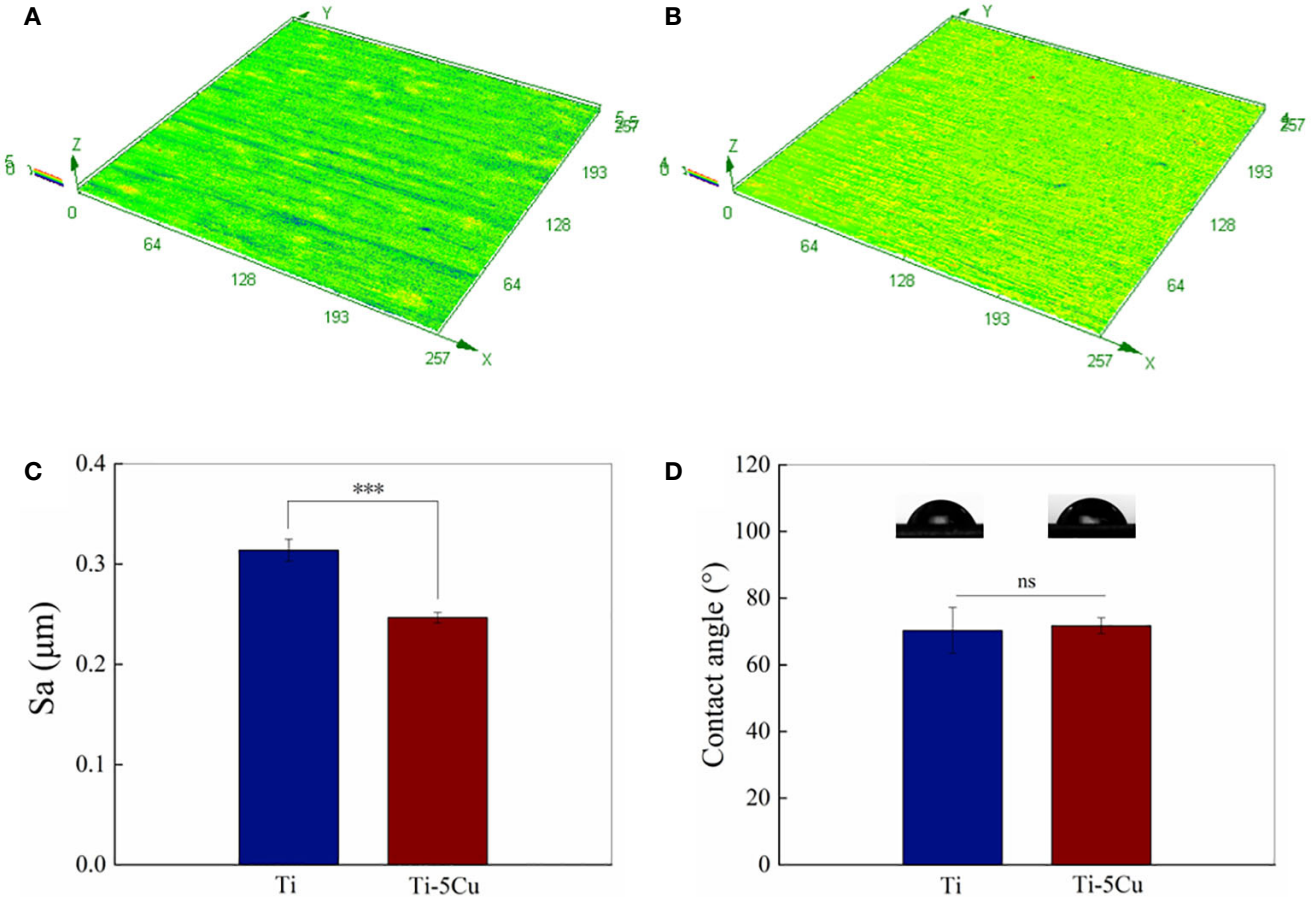
Surface roughness and hydrophilicity. **(A)** 3D image surface roughness of Ti; **(B)** 3D image surface roughness of Ti-5Cu; **(C)** Statistical analysis of surface roughness (Sa); **(D)** Contact angles of Ti and Ti-5Cu alloy (n = 4; ***p < 0.001; ns, no significant difference).

The average 3D surface roughness of Ti and Ti-5Cu alloy is shown in **Figures 2A–C**. Ti-5Cu alloy presented a much smoother surface. The Sa value of Ti-5Cu is 0.2465 ± 0.0046 μm, which is significantly lower than that of Ti that is 0.3138 ± 0.0096 μm, indicating that the addition of Cu in pure Ti decreased its surface roughness. However, as shown in **Figure 2D**, the contact angle of Ti-5Cu alloy is 71.8 ± 2.0° which shows no significant difference compared with Ti of 70.3 ± 6.0°.

### 3.3 Cu^2+^ release and cytotoxicity

**Figure 3:**
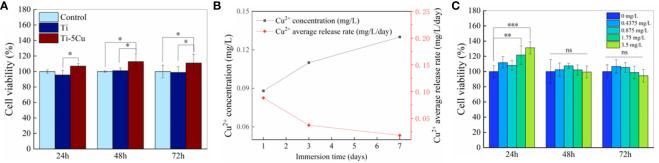
Cu^2+^ release and cytotoxicity. **(A)** The cell viability of RAW264.7 after co-cultured with Ti and Ti-5Cu alloy at 24, 48, and 72 h, respectively. **(B)** Concentration and average release rate of Cu^2+^ in Ti-5Cu alloy immersed in DMEM medium. **(C)** The cell viability of RAW264.7 cultured in different concentration of Cu^2+^ culture medium at 24, 48, and 72 *h*, respectively (n = 4; *p < 0.05, **p < 0.01, and ***p < 0.001; ns, no significant difference).

The cytotoxicity of Ti and Ti-5Cu alloy to RAW264.7 cells is shown in **Figure 3A**. The RAW264.7 cell viability of Ti-5Cu alloy was much higher than that of Ti group and control group; the differences had statistically significance at 48 and 72 *h* (p < 0.05). The results indicated that Ti-5Cu alloy has no cytotoxicity to RAW264.7 cells.

To increase the concentration of Cu^2+^, six pieces of Ti or Ti-5Cu alloy were co-cultured with RAW264.7 in a six-well plate. The ICP-MS was used to detect the concentration and average release rate of Cu^2+^ in Ti-5Cu alloy immersed in DMEM medium. As the results showed in **Figure 3B**, the concentration of Cu^2+^ increased gradually within 7 days, the concentration in the first 7 days was 0.133 mg/L. However, the average release rate of Cu^2+^ was 0.088 mg/L/day on the first day and then decreased to 0.019 mg/L/day on the seven day.

The cytotoxicity of Cu^2+^ at the concentration detected above to RAW264.7 was measured by CCK-8. As **Figure 3C** shows, the Cu^2+^ concentration at 1.75 and 3.5 mg/L significantly increased the cell viability of RAW264.7 at 24 *h* (p < 0.01), and then, the cell viability of RAW264.7 in different concentration of Cu^2+^ group had no significant difference compared with the group of 0 mg/L at 48 and 72 *h*. These results indicated that the Cu^2+^ concentration at the range of 0–3.5 mg/L had no obvious cytotoxicity to RAW264.7 cells.

### 3.4 Ti-5Cu alloy promoted the M2 polarization of macrophages

#### 3.4.1 The expressions of surface makers

**Figure 4:**
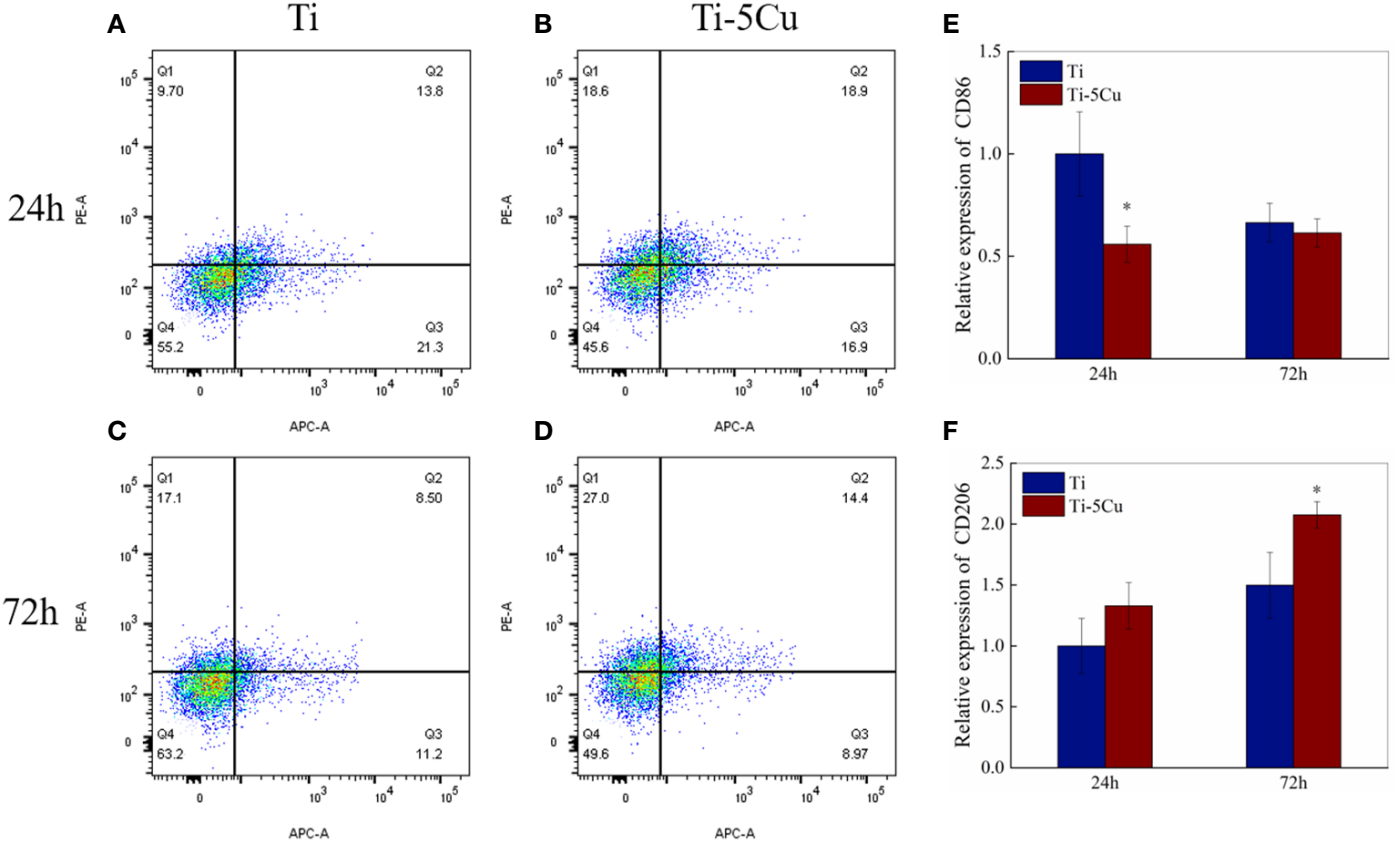
The expressions of CD86 and CD206 in RAW264.7 cells co-cultured with Ti and Ti-5Cu alloy at 24 and 72 *h* by flow cytometry and RT-qPCR. **(A)** Ti at 24 *h*; **(B)** Ti-5Cu at 24 *h*; **(C)** Ti at 72 *h*; **(D)** Ti-5Cu at 72 *h*; **(E)** the gene expressions of CD86; **(F)** the gene expressions of CD206 (n = 3; *p < 0.05).

The flow cytometry test was used to detect the expressions of CD86 and CD206 in RAW264.7 cells co-cultured with Ti and Ti-5Cu alloy at the time point of 24 and 72 *h*. As **Figures 4A–D** show, the upper left quadrant (Q1) represents APC−PE+ cells (CD86−CD206+), and the lower right quadrant (Q3) represents APC+PE− cells (CD86+CD206−). At 24 *h*, the percentage of CD86 expression in Ti-5Cu alloy is 16.9%, which is much lower than that in Ti (21.3%); instead, the percentage of CD206 expression in Ti-5Cu alloy (18.6%) is much higher than that in Ti (9.7%). At 72 *h*, similar results were also obtained. The percentage of CD206 expression in Ti-5Cu alloy showed 27.0%, higher than that in Ti (17.1%), whereas the percentage of CD86 expression in Ti-5Cu alloy showed 8.97%, which is much lower than that in Ti (11.2%). The gene expressions of CD86 and CD206 were detected by RT-qPCR; as the results showed in **Figures 4E, F**, Ti-5Cu alloy significantly decreased the expressions of CD86 at 24 *h* and increased the expressions of CD206 at 72 *h* when compared with Ti (p < 0.05).

#### 3.4.2 The expressions of inflammatory cytokines

**Figure 5:**
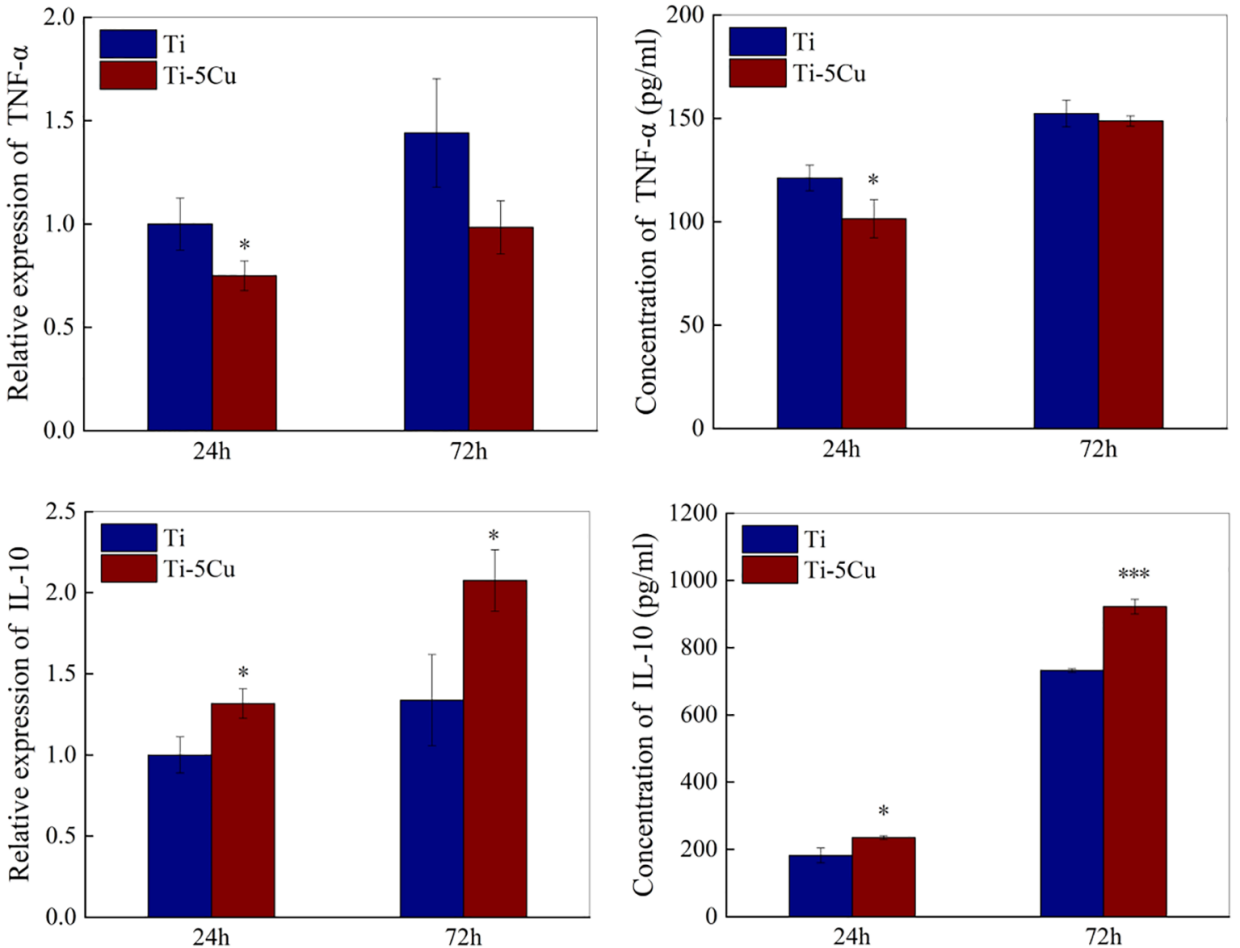
The gene and protein expressions of inflammatory cytokines of RAW264.7 cells after co-cultured with Ti and Ti-5Cu alloy at 24 and 72 *h* by RT-qPCR and ELISA (n = 3; *p < 0.05 and ***p < 0.001).

The gene and protein expressions of pro-inflammatory cytokines (TNF-α) and anti-inflammatory cytokines (IL-10) were measured by RT-qPCR and ELISA. As the results showed in **Figure 5**, the gene expressions of TNF-α in Ti-5Cu alloy decreased compared with Ti and had statistically difference at 24 *h* (p < 0.05). Meanwhile, Ti-5Cu alloy significantly increased the gene expressions of IL-10 at 24 and 72 *h* (p < 0.05).

The concentrations of TNF-α and IL-10 secreted by RAW264.7 cells at 24 and 72 *h* were assessed by ELISA. As shown in **Figure 5**, the concentration of TNF-α in Ti-5Cu was decreased compared with Ti, and the difference had statistically significance at 24 *h* (p < 0.05). However, Ti-5Cu alloy significantly promoted the IL-10 expression at 24 *h* (p < 0.05) and 72 *h* (p < 0.001).

### 3.5 Ti-5cu alloy increased the expressions of osteogenic cytokines in RAW264.7 cells

**Figure 6:**
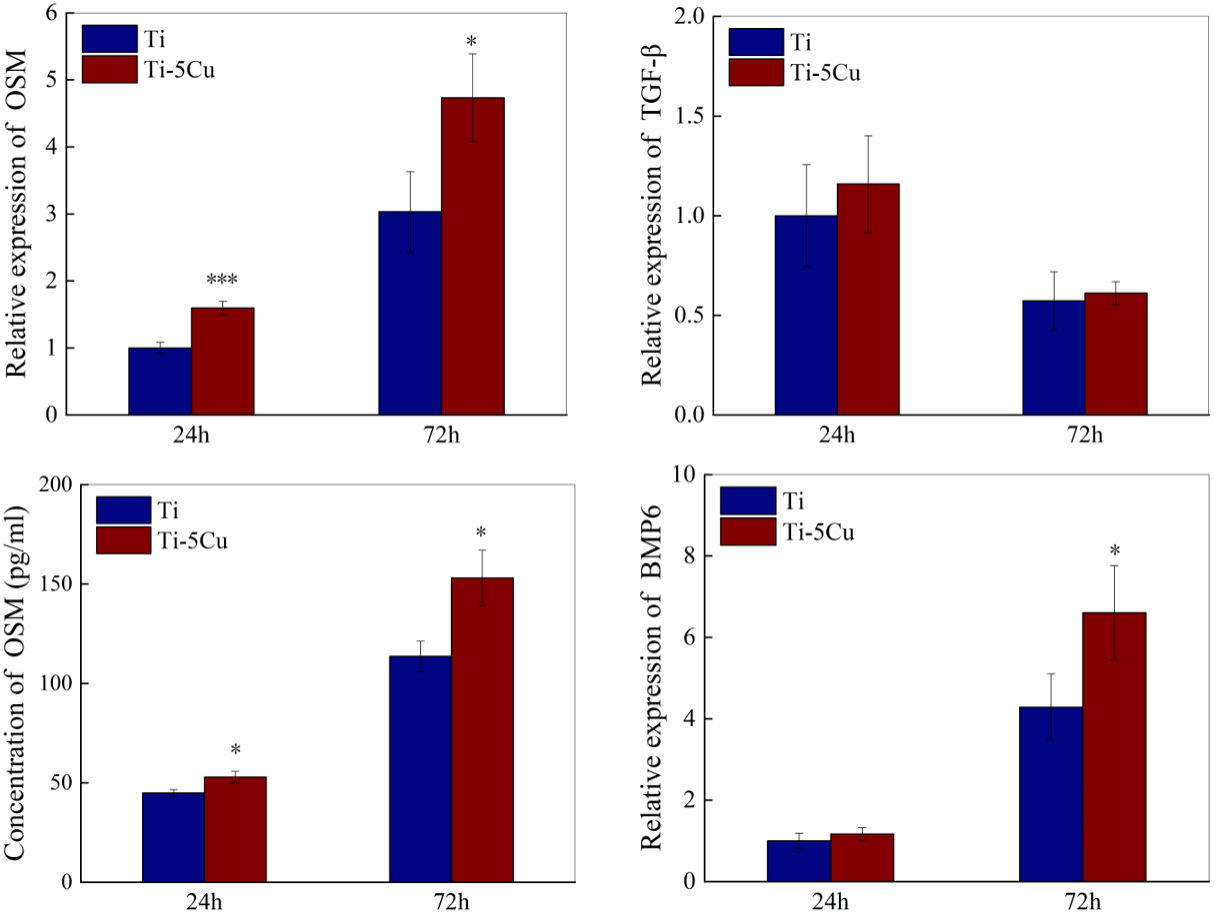
The gene and protein expressions of osteogenic cytokines in RAW264.7 cells after co-cultured with Ti and Ti-5Cu alloy at 24 and 72 *h* by RT-qPCR and ELISA (n = 3; *p < 0.05, ***p < 0.001).

The gene and protein expressions of osteogenic cytokines secreted by RAW264.7 cells after co-cultured with Ti or Ti-5Cu alloy were measured by RT-qPCR and ELISA. As the results showed in **Figure 6**, Ti-5Cu alloy significantly promoted the OSM gene expressions at 24 *h* (p < 0.001) and 72 *h* (p < 0.05). The gene expressions of TGF-β and BMP6 also increased in Ti-5Cu alloy, but there was no statistically difference in TGF-β at both time points and BMP6 at 24 *h*. ELISA results showed that Ti-5Cu alloy significantly increased the protein expression of OSM at 24 and 72 *h* (p < 0.05), which were consistent with RT-qPCR results.

### 3.6 The extracts from Ti-5Cu alloy enhanced the M2 polarization of RAW264.7 cells

**Figure 7:**
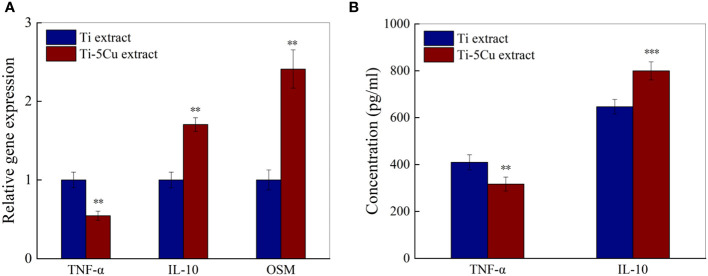
The gene and protein expressions of inflammatory and osteogenic cytokines in RAW264.7 cells after cultured with Ti and Ti-5Cu extracts for 72h. **(A)** The gene expressions of RAW264.7 cells by RT-qPCR; **(B)** The protein expressions of RAW264.7 cells by ELISA(n = 3; **p < 0.01 and ***p < 0.001).

The gene and protein expressions of inflammatory cytokines and osteogenic cytokines in RAW264.7 cells after cultured with Ti and Ti-5Cu extracts for 72 *h* were measured by RT-qPCR and ELISA. As the results showed in **Figure 7A**, compared with Ti, Ti-5Cu extract significantly decreased the gene expression of TNF-α and increased the expression of IL-10 (p < 0.01). In addition, the gene expression of osteogenic cytokine OSM in Ti-5Cu extract is significantly higher than Ti extract (p < 0.01). The protein expression results are shown in **Figure 7B**; Ti-5Cu extract significantly decreased the concentration of TNF-α (p < 0.01) and increased the concentration of IL-10 (p < 0.001). These results indicated that the Ti-5Cu extract promoted the M2 polarization of RAW264.7 and the gene expression of OSM.

### 3.7 Behaviors of MC3T3-E1 cells cultured with conditioned medium

#### 3.7.1 Conditioned medium derived from Ti-5Cu alloy promoted proliferation of MC3T3-E1 cells

**Figure 8:**
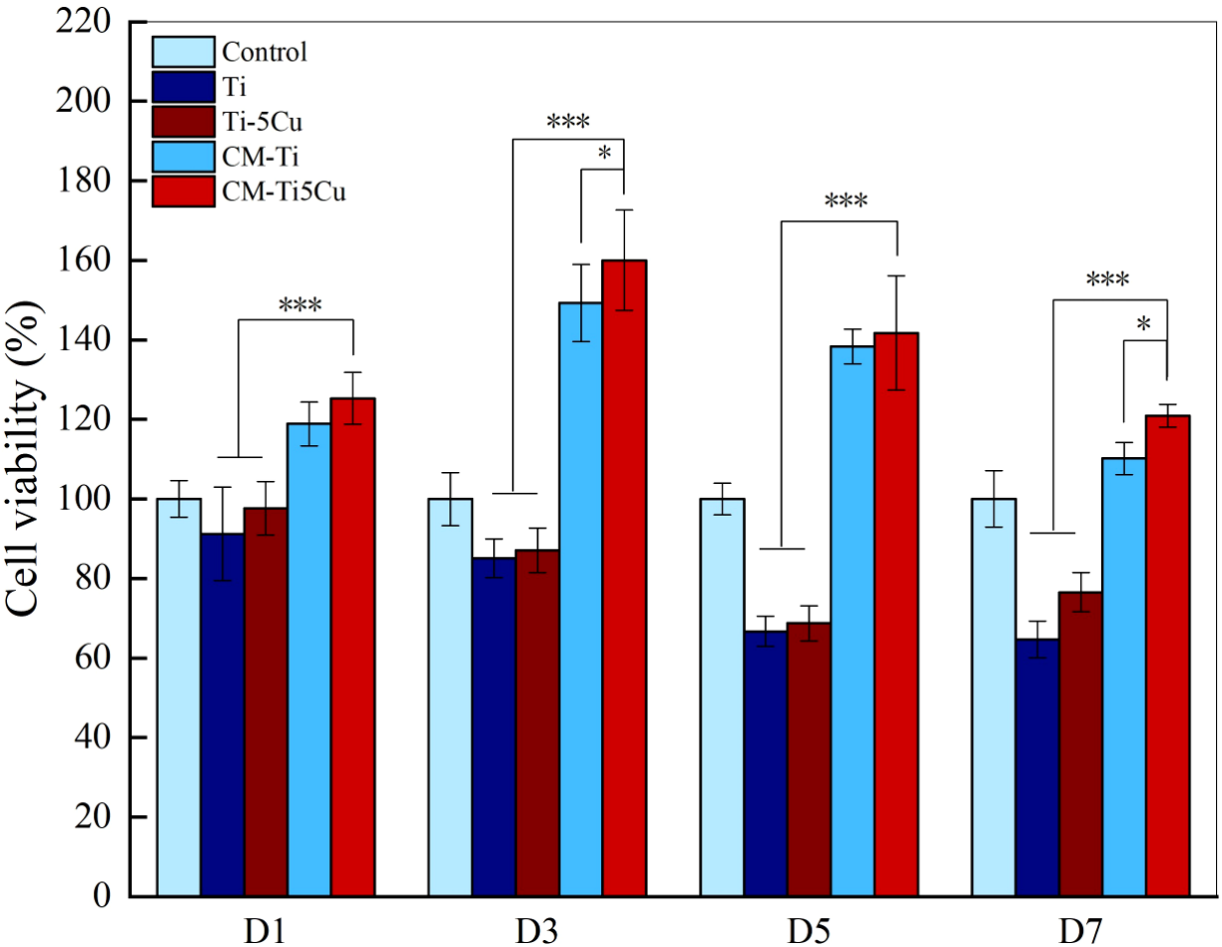
The cell viability of MC3T3-E1 cells cultured with conditioned medium and material extracts at D1, D3, D5, and D7, respectively (n = 4; *p < 0.05, ***p < 0.001).

The effects of conditioned medium on the proliferation of MC3T3-E1 cells were detected by CCK-8. As the results showed in **Figure 8**, CM-Ti5Cu group significantly promoted the cell viability of MC3T3-E1 at all time points compared with Ti and Ti-5Cu extracts (p < 0.001). Moreover, on days 3 and 7, the cell viability of MC3T3-E1 in CM-Ti5Cu group was much higher than that in CM-Ti group (p < 0.05).

#### 3.7.2 Conditioned medium derived from Ti-5Cu alloy accelerates the osteogenic differentiation of MC3T3-E1 cells

**Figure 9:**
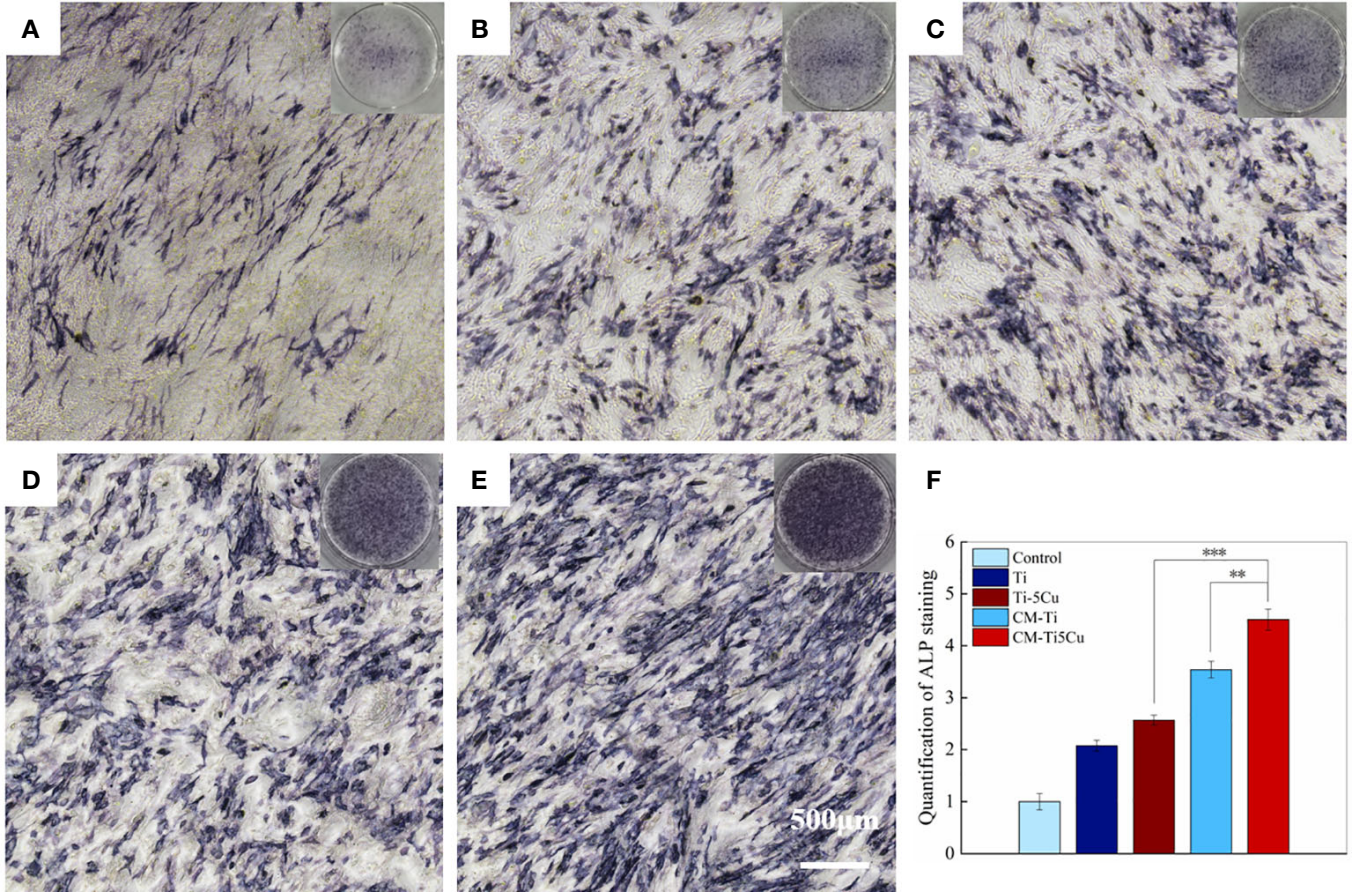
The ALP staining of MC3T3-E1 cells after cultured with conditioned medium and material extracts for 7 days. **(A)** Control group; **(B)** Ti extract group; **(C)** Ti-5Cu extract group; **(D)** CM-Ti group; **(E)** CM-Ti5Cu group; **(F)** quantification statistical analysis of ALP staining (n = 3; **p < 0.01 and ***p < 0.001).

The ALP staining results were shown in **Figure 9**. The ALP activity can be indicated by insoluble regions stained with dark blue or blue purple in the image. It can be seen that there were more ALP staining areas in CM-Ti5Cu compared with CM-Ti and other groups, and the quantification of ALP staining proved that the differences had statistically significance (p < 0.001).

**Figure 10:**
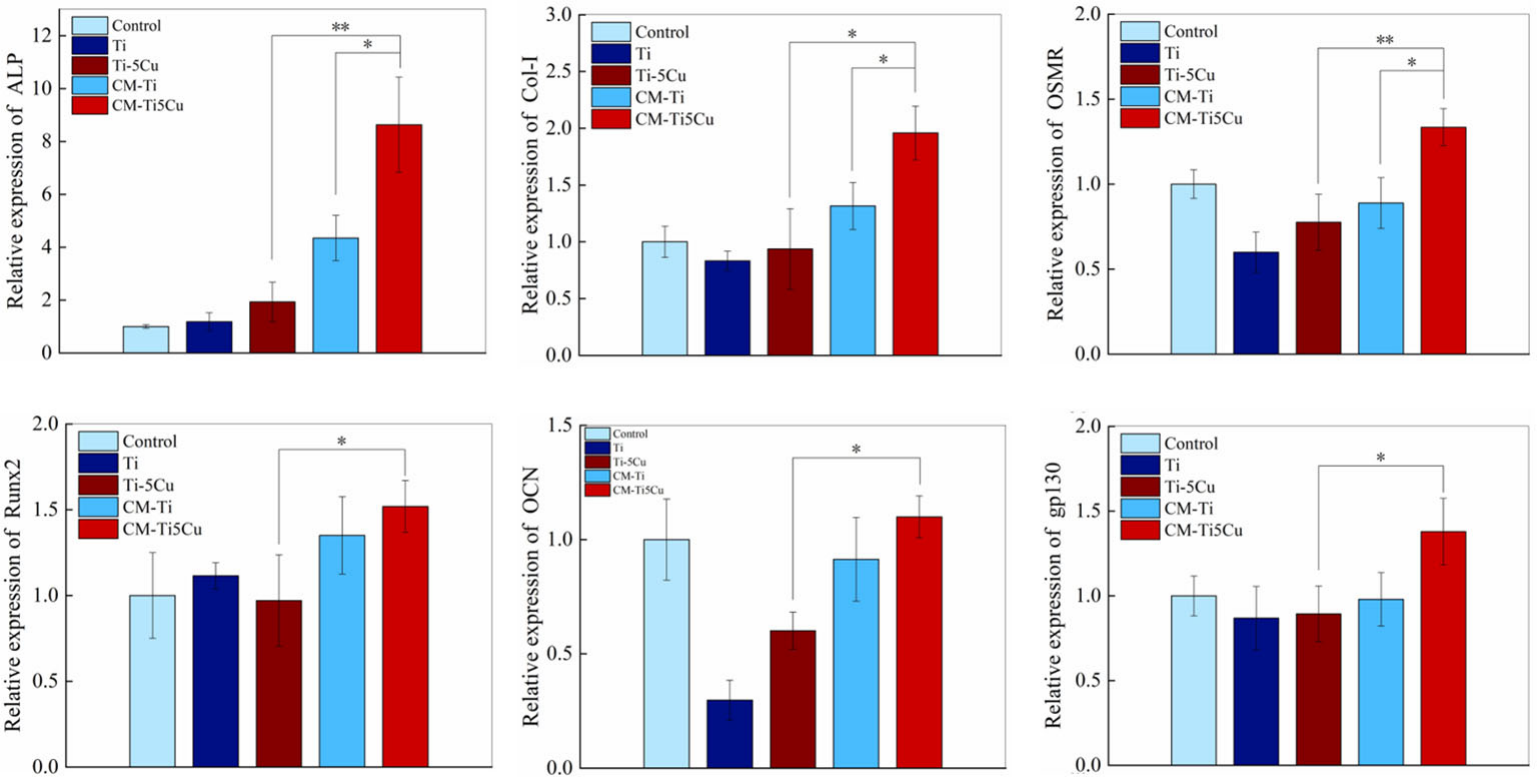
The expressions of osteogenic genes and OSM-related receptors genes of MC3T3-E1 cells after cultured with conditioned medium and material extracts for 7 days by RT-qPCR (n = 3; *p < 0.05 and **p < 0.01).

The expressions of osteogenesis genes (ALP, COL-I, Runx2, and OCN) and OSM signaling pathway–related genes (OSMR and gp130) in MC3T3-E1 cells were tested by RT-qPCR. As shown in **Figure 10**, CM-Ti5Cu significantly increased the expressions of ALP, COL-I, Runx2, OCN, OSMR, and gp130 genes compared with Ti or Ti-5Cu extracts (p < 0.05). In addition, the gene expressions of ALP, COL-I, and OSMR in CM-Ti5Cu were significantly higher than that in CM-Ti (p < 0.05).

### 3.8 OSM secreted by RAW264.7 cells promoted the osteogenic differentiation of MC3T3-E1 cells through acting on OSMR/gp130 receptors

In previous experiment, we verified that Ti-5Cu alloy significantly promoted gene and protein expressions of OSM in RAW264.7 cells at 24 and 72 *h*. Therefore, to test the specific osteogenesis mechanism of MC3T3-E1 cells cultured in conditioned medium derived from Ti-5Cu alloy, we used siRNA to knock down the expression of OSM gene in RAW264.7 cells and detected the effects of the conditioned medium on MC3T3-E1 osteogenic differentiation.

**Figure 11:**
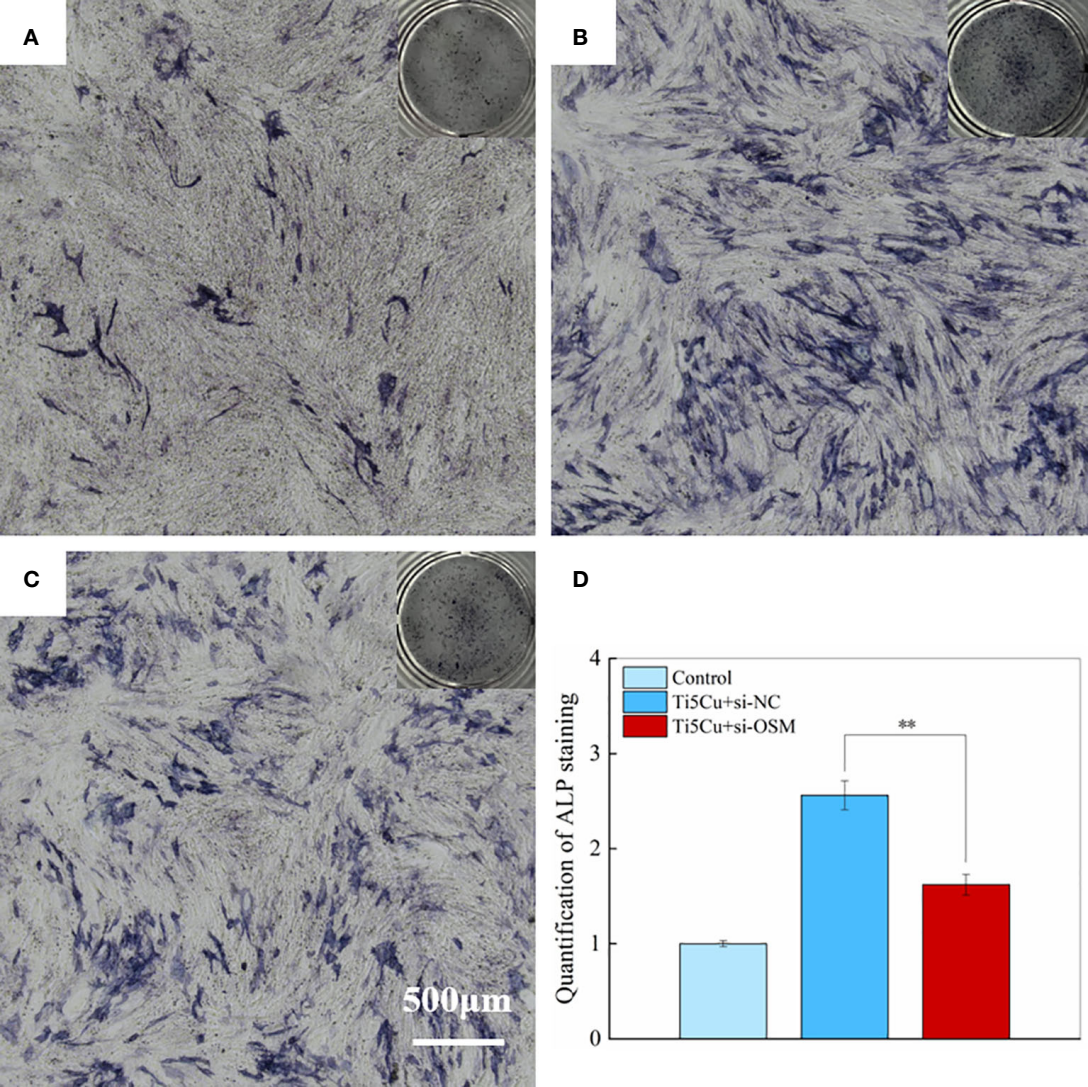
The ALP staining of MC3T3-E1 cells after cultured with si-NC and si-OSM conditioned medium for 7 days. **(A)** Control group; **(B)** Ti5Cu + si-NC group; **(C)** Ti5Cu + si-OSM group; **(D)** quantification statistical analysis of ALP staining (n = 3; **p < 0.01).

We effectively decreased the expression of OSM gene in RAW264.7 cells, as the result showed in the supplementary materials, the knockdown efficiency was about 60%. The results of ALP staining were shown in **Figure 11**; fewer ALP staining areas were showed in MC3T3-E1 cells after silencing the OSM gene in RAW264.7 cells compared with that in the NC group, and the difference was statistically significant (p < 0.01).

**Figure 12:**
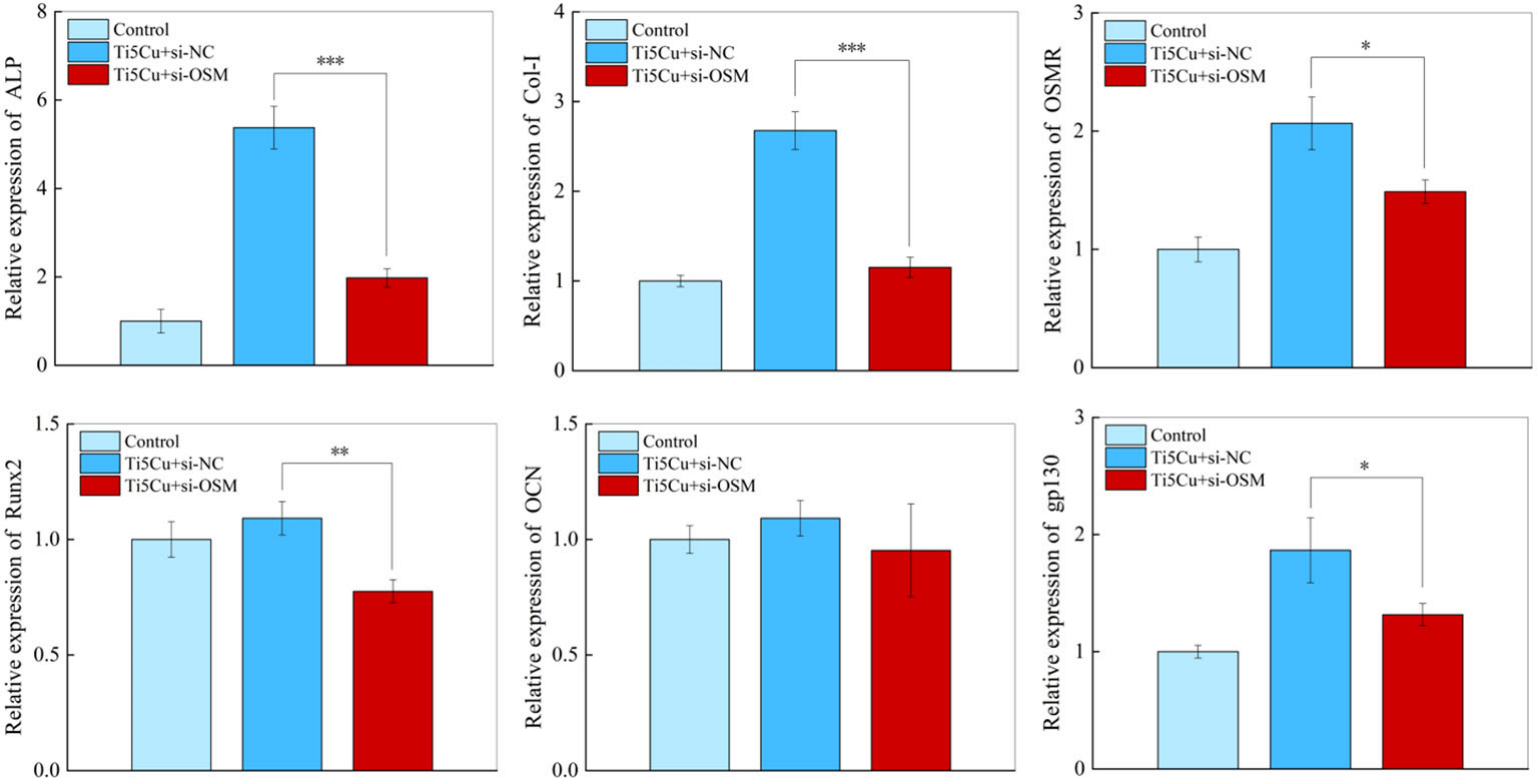
The expressions of osteogenic genes and OSM-related receptors genes of MC3T3-E1 cells after cultured with si-NC and si-OSM conditioned medium for 7 days by RT-qPCR (n = 3; *p < 0.05, **p < 0.01, and ***p < 0.001).

The RT-qPCR results (**Figure 12**) showed that the ALP, COL-I, and Runx2 gene expressions in MC3T3-E1 cells were significantly decreased after silencing the OSM gene in RAW264.7. At the same time, the expressions of OSM signaling pathway–related genes OSMR and gp130 were also decreased in MC3T3-E1 cells, and the differences were statistically significant (p < 0.05).

## 4 Discussion

Titanium alloy has been widely used in oral implants and orthopedic implants due to its good mechanical and biological properties. Cu is an essential trace element in human body and has certain antibacterial activity; so in recent years, Ti-Cu alloy has caused much attention for its good antibacterial ability, excellent corrosion resistance, and bone formation ability (14, 26); they are promising to be applied in orthopedic or oral implants for various bone defects. However, as foreign bodies, most implants tend to inevitably attract the attention of immune cells after implantation. Macrophages are chief participants in host inherent immune response; they can polarize into either pro-inflammatory M1 phenotype or anti-inflammatory M2 phenotype depending on the stimulus in the environment (4). However, there are few studies on the regulation of macrophage polarization in terms of Ti-Cu alloy.

Ti and Ti-5Cu alloy used in this experiment were fabricated by 3D printing technology that was also known as additive manufacturing; it is based on digital documents, information technology, precision machinery, and materials science. The data obtained by CT scanning can be converted into 3D images, and then, the implant size and shape are designed for the bone defects in 3D images with computer assistance. After the design is completed, the obtained data are imported into 3D printers, and the 3D images are processed by selective laser melting technology to print implant molds that meet clinical needs (2). In this study, the SEM results showed that the β phase of Ti element in both materials is completely transformed into α phase, which is caused by the high-temperature gradient during the 3D printing process (27). In the molten pool of the liquid phase, the Cu element was fully diffused, and most of the Cu element was solid-dissolved in the α grains after cooling (10). Furthermore, the addition of Cu did not change the phase composition of Ti, both of which were mainly α-Ti phase as the XRD results showed above. In the meantime, the EDS results showed that the 3D printed Ti-5Cu alloy was composed of Ti and Cu, and Cu was uniformly distributed on the surface of Ti-5Cu alloy.

In addition, metal ions in biomaterials will diffuse to surrounding tissues after implantation; so to ensure long-term stability, implants must have excellent corrosion resistance and strength. Ren et al. (28) have studied the influence of different Cu content on the corrosion resistance of TiCu alloy. The results showed that the corrosion resistance of TiCu alloy is better than that of Ti, and the corrosion resistance of TiCu alloy is improved with the increase of Cu content. Cu’s strong solid-state solution strengthening and dispersion strengthening ability endow TiCu alloys with high strength, which is beneficial for load-bearing applications of TiCu alloys. Zhang et al. (29) also found that the hardness and corrosion resistance of TiCu alloys increased with the increase of Cu content. The Cu content in the TiCu alloy is both below 10% in the above two studies.

Studies have shown that the surface roughness and hydrophilicity of materials affect macrophage polarization (30). Kosoff et al. (31) investigated the effects of surface morphology and hydrophilicity on macrophage phenotype; the results showed that the expression of M2-related genes was increased in macrophages cultured on smooth surfaces with higher hydrophilicity. Another study also revealed that macrophages cultured on higher wettability surface presented greater anti-inflammatory properties (8). In this study, although the hydrophilicity of Ti and Ti-5Cu showed no difference, we found that surface roughness of Ti-5Cu was much lower than that of Ti, which may indicate that the addition of Cu in pure Ti provided materials with much smoother surface, thus contributing to the M2 polarization of RAW264.7 cells.

Cu is an essential element for maintaining the homeostasis of body, which is usually obtained from daily diet or water and the recommended amount for adults is 2–3 mg every day (32). Excessive Cu intake or deficiency will result in many diseases, like Wilson disease and Alzheimer disease (33). It has been proved that Cu^2+^ promoted the proliferation of RAW264.7 cells when the concentration was at the range of 0.5–4 ppm and had obvious cytotoxicity when the concentration exceeded 16 ppm (34). It is speculated that the reason why Cu promotes cell proliferation at low concentration may be related to that Cu, as an essential element and a co-factor of many enzymes in cells, participates in cell metabolism that includes cell energy production, signal transduction, proliferation, and oxidation (35). Nevertheless, the excessive concentration of Cu ions is more likely to result in copper metabolism imbalance, thus exhibiting certain cytotoxicity and leading to the occurrence of disease (36).

To study the cytocompatibility of materials, the CCK-8 method was used to evaluate the cytotoxicity of Ti and Ti-5Cu to RAW264.7 cells. The results showed that Ti-5Cu alloy had no cytotoxicity to RAW264.7 cells and promoted cell proliferation at all time points. However, in one of our previous studies, it was found that the release concentration of Cu^2+^ in TC4-5Cu alloy in 24-well plate was only less than 10 μg/L in the first 7 days (25). Therefore, to study the effect of higher concentration of Cu^2+^ on RAW264.7 polarization, we chose the co-culture method by placing six pieces of materials into six-well plates to increase the concentration of Cu^2+^. We used ICP-MS to detect the Cu^2+^ concentration and the average release rate in Ti-5Cu alloy at 1, 3, and 7 days, and the results showed that the total Cu^2+^ concentration at 7 days was 0.133 mg/L, which was much lower than the recommended amount. Studies have shown that, when the concentration of Cu^2+^ is equal to or higher than 200 μM, it will lead to a significant decrease in the metabolic activity of THP-1 cells, and when the concentration is higher than 500 μM, it will lead to a decrease in the DNA concentration of the cells. The decrease in cell viability caused by high concentrations of Cu^2+^ is related to the damage of mitochondrial activity (37). In this study, according to **Figure 3C**, it was shown that Cu^2+^ concentration released in 1 and 3 days has no cytotoxicity to RAW264.7 cells; then, we studied the effects of materials on macrophage polarization.

However, the biosafety of Cu^2+^ continuously released after implantation of Cu-containing biomaterials also needs to be considered. Fan et al. (25) investigated the release concentration and release rate of Cu^2+^ from a gradient Cu-bearing titanium alloy over 28 days. They found that Cu^2+^ was continuously released over 28 days; the highest concentration was only 18.73 ± 0.87 μg/L; at the same time, the release rate of Cu^2+^ gradually decreased, which was consistent with our results. Liu et al. (38) fabricated a TiCu alloy containing 5 wt% Cu into a cylinder implant with a size of ϕ3.6 × 8 mm. They compared the anti-infection ability and inflammatory responses of Ti and TiCu implants after implantation through *in vivo* animal experiments. The results revealed that, after 3 months, the amount of Cu^2+^ in the bone tissue around the TiCu implant was 0.55 μg/g, which is slightly higher than that around the Ti implant (0.45 μg/g). This increase of Cu^2+^ concentration did not cause significant inflammation. Instead, they found more inflammatory cell infiltrations in the soft tissue around the Ti implant. These results indicate that TiCu implants can effectively inhibit the inflammatory response of gingival tissue. Moreover, the amounts of inflammatory cytokines in the serum of the animals were within the normal range after 3 months of the implantation, which indicated that TiCu implant had good biosafety.

TNF-α and CD86 were highly expressed in M1-type macrophages, whereas IL-10 and CD206 were highly expressed in M2-type macrophages. Therefore, to detect the effects of Ti or Ti-5Cu alloy on RAW264.7 polarization at 24 and 72 **h**, the relative mRNA expressions of TNF-α, IL-10, CD86, and CD206 genes were measured by RT-qPCR. Because of the same species as MC3T3-E1 cells and as one of the common inflammatory model cells, the RAW264.7 cells were used in this experiment (39). As it can be seen from the results, Ti-5Cu alloy significantly promoted the M2 polarization of RAW264.7 cells compared with Ti, presented by the decreased expression of M1-related cytokines (TNF-α and CD86) and the increased expression of M2-related cytokines (IL-10 and CD206) both at 24 and 72 h. Many scholars have also studied the effect of copper containing biomaterials on the polarization of macrophages. An *in vivo* study revealed that, by adding 5% Cu into porous bioactive glass, the Cu-MBG group resulted in more M2 macrophage infiltration, manifested by the upregulation of CD163 and the downregulation of CCR7, suggesting that the material may regulate M2 polarization of macrophages (40). Lin et al. (34) prepared bioactive glass ceramics scaffolds containing 5% Cu and found that, compared with the Bioactive Glass Ceramics (BGC) group, Cu-BGC extracts could polarize macrophages to M2 type, presented by the downregulation of iNOS and TNF-α and upregulation of CD206 and IL-10 at days 1 and 3; they also studied the releasing concentration of Cu^2+^ from Cu-BGC in Tris-HCL solution; the concentration of Cu^2+^ at days 1 and 3 was about 0.5 mg/L. Furthermore, their results showed that Cu^2+^ could promote the transformation of macrophages into an anti-inflammatory phenotype when the concentration was lower than 16 mg/L. In this study, we found that, compared with Ti, Ti-5Cu–derived extract promoted the M2 polarization of RAW264.7 cells, which was manifested by the decreased expression of pro-inflammatory cytokine TNF-α and the increased expression of anti-inflammatory cytokine IL-10. Therefore, we speculated that Cu^2+^ released from Ti-5Cu alloy in this experiment may also be another reason to promote M2 polarization of RAW264.7 cells. The concentration of Cu^2+^ also affects the polarization phenotype of macrophages (41). Macrophages were more likely to polarize into the M2 phenotype when Cu^2+^ is at low concentration (1–5 μM), but with the increase of Cu^2+^ concentration (> 12 μM), macrophages tended to be more polarized to the M1 phenotype (42, 43). In our previous work (44), we used flow cytometry to compare the effects of TC4 alloy and TC4-5Cu alloy on RAW264.7 polarization, and it was found that there was no significant difference at 24 and 72 h on the expression of CD86 and CD206.We speculated that it was because the releasing concentration of Cu^2+^ was too low and the addition of Cu did not change the surface roughness of TC4 alloy. Although the culture conditions were same as the study just mentioned, another experiment found that Ti-5Cu was more inclined to polarize RAW264.7 cells into M2 type compared with TC4, showing an increase in CD206 and a decrease in CD86. The reason for the difference between the two studies may be that the experimental material of the latter was porous material that could release more Cu^2+^ (45). Tercero et al. (37) investigated the immune regulation effects of different concentrations of Cu^2+^ on macrophages. Their results showed that, when the concentration of Cu^2+^ was less than or equal to 10 μM (0.64 mg/L), it was more prone to polarize macrophages to M2 phenotype; in contrast, when the concentration was greater than 100 μM (6.45 mg/L), it could significantly polarize the macrophage to M1 phenotype. The anti-inflammatory effect of Cu^2+^ may be related to the activation of the Hypoxia Inducible Factor (HIF) signaling pathway (34). Cu can also act as a co-factor of superoxide dismutase (SOD) to play an antioxidant role and reduce the production of intracellular reactive oxygen species (ROS), thus inhibiting the release of pro-inflammatory cytokines (46). Moreover, SOD can convert superoxide anion $\left(O_{2}^{\cdot -}\right)$to H_2_O_2_ to increase the production of H_2_O_2_. This SOD-mediated production of H_2_O_2_ can promote M2 polarization of macrophages through STAT6 signaling pathway (47). Moreover, Cu^2+^ can activate arginase, which inhibits NO production and the release of pro-inflammatory cytokine while enhancing PGE_2_ production in macrophages (48). The increase of PGE_2_ can activate the activity of its membrane receptors EP2 and EP4, upregulated the expressions of the downstream genes cAMP and CREB and further polarized macrophages to M2 phenotype by promoting the release of anti-inflammatory factors (49). Although the specific anti-inflammatory mechanism of Cu^2+^ at certain concentration was not studied in this experiment, it has been proved that Ti-5Cu alloy promotes the M2 polarization of RAW264.7 cells, which is beneficial to tissue regeneration.

Macrophage-derived cytokines play an important role in osteogenic differentiation of osteoblasts (18). Pro-regenerative cytokines produced by M2 macrophages, such as TGF-β and vascular endothelial growth factor, can accelerate the osteogenic differentiation of mesenchymal stem cells (MSCs) (50). A nanoparticle-mediated M2 polarization of macrophages promotes the secretion of IL-10, which can enhance the osteogenesis of MSCs (51). Cytokines mainly act on their corresponding receptors and then signal osteoblasts to promote osteogenesis (52). For example, BMP2 can activate the phosphorylation of drosophila mothers against decapentaplegic protein (SMAD1) by acting on BMP receptors, promote the nuclear ectopic of RUNX2, and then upregulate the gene expressions of ALP and osteocalcin in pre-osteoblasts (53). In addition, extracellular vesicles (EVs) secreted by macrophages can also affect bone formation. EVs derived from M0 or M2 macrophages can promote bone repair, whereas M1 EVs inhibit bone repair (54). In this experiment, we detected the gene expressions of osteogenesis cytokines in RAW264.7 cells co-cultured with Ti-5Cu alloy at 24 and 72 h. As shown in the results, Ti-5Cu alloy promoted the OSM, TGF-β, and BMP6 gene expression of RAW264.7. Then, we verified the conditioned medium derived from Ti-5Cu alloy co-cultured with RAW264.7 on the proliferation and osteogenic differentiation of MC3T3-E1 cells. The MC3T3-E1 cells have the ability to self-renew and differentiate into osteoblasts, so it has been used as a model to study osteogenic capacity *in vitro (* 55). To eliminate the influence of the ions extracted from materials on the results, we prepared material extracts without RAW264.7 cells under the same culture conditions. The CCK-8 results demonstrated that the conditioned medium derived from RAW264.7 cells co-cultured with Ti and Ti-5Cu alloy promoted MC3T3-E1 proliferation; especially at day 7, the CM-Ti5Cu presented excellent cytocompatibility. It has been proved that OSM derived from macrophages can promote the proliferation of cardiomyocytes (56). Therefore, we speculated that OSM and other cytokines secreted by RAW264.7 cells in the CM-Ti5Cu played an important role in promoting MC3T3-E1 cell proliferation. The results of RT-qPCR and ALP staining showed that the conditioned medium derived from Ti-5Cu alloy could significantly promote the osteogenic differentiation of MC3T3-E1 cells. The osteogenesis ability of OSM has also been reported in other biomaterials; a study found that CS bioactive ceramics could regulate macrophage polarized to M2 type; and the OSM secreted by Bone Marrow Derived Macrophages (BMDM) promoted the osteogenic differentiation of BMSCs (57). However, different from the indirect co-culture method in this study, another study directly co-cultured human monocyte-derived macrophages and human MSCs (hMSCs), finding that a carbon nanohorn could promote osteogenic differentiation of hMSCs by activating macrophages to secrete OSM (58).

To verify whether it is OSM secreted by RAW264.7 cells that promoted osteogenic differentiation of MC3T3-E1 cells, we used siRNA to knock down the expression of OSM gene in RAW264.7 and prepared a conditioned medium to detect its effect on MC3T3-E1 osteogenic differentiation. At the same time, it is known that OSM mainly conducts signal transduction through β-receptor glycoprotein 130 (gp130). Only after OSM binding to its specific receptor OSMR, a dimer signal transduction complex containing gp130 can be formed to activate gp130-mediated signal transduction (21). Hence, we also detected the gene expressions of OSMR and gp130 that were associated with OSM-related signaling pathway. As shown in the results, the osteogenic differentiation ability of MC3T3-E1 was decreased after the OSM gene was silenced, presented by the decreased gene expression of ALP, COL-I, and Runx2, which were consistent with the ALP staining results. Moreover, the expression of OSMR and gp130, upstream molecules of OSM-related signaling pathway, was also significantly decreased. These results suggest that OSM secreted by RAW264.7 indeed promotes osteogenic differentiation of MC3T3-E1 by acting on OSMR and gp130 receptor. Several scholars have studied the osteogenic differentiation mechanism of OSM on MC3T3-E1 cells. Zheng et al. (59) revealed that OSM promoted the osteogenic differentiation of MC3T3-E1 through MMP-1. It has also been found that Notch signaling pathway plays a negative regulatory role in OSM promoting MC3T3-E1 osteogenic differentiation (24). Meanwhile, OSM could promote regulation of the osteogenic differentiation of dental pulp stem cells through STAT3 pathway (60). In addition, OSM has been proved to be a stimulator of WNT16, which can stimulate bone formation by upregulating the WNT signaling pathway (61). However, the specific signal transduction mechanism of OSM in this study was not analyzed, which needs to be further clarified.

Immune response regulated by biomaterials may be a potential therapeutic approach to promote bone formation. On the basis of the similar corrosion resistance with Ti, good antimicrobial properties, and biocompatibility, Ti-5Cu alloy can also promote the osteogenic differentiation of pre-osteoblasts through promoting the M2 polarization of macrophages, which indicates that Ti-5Cu alloy has favorable bone immune regulatory properties. It may become a promising biomaterial in the field of dental implants and orthopedic implants for maxillofacial defects.

## 5 Conclusions

In this study, we fabricated Ti-5Cu alloy through 3D printing technology; the addition of 5 wt% Cu had little effect on the hydrophilicity of materials, but the Ti-5Cu alloy presented much smoother surface compared with Ti. We also demonstrated that Ti-5Cu alloy had no cytotoxicity to RAW264.7 cells. In macrophage polarization, the RAW264.7 is more likely to be polarized to M2 phenotype after co-cultured with Ti-5Cu alloy. Moreover, OSM is an important cytokine secreted by RAW264.7 cells that promotes osteogenic differentiation of MC3T3-E1 cells. The mechanism may be that OSM acts on OSMR/gp130 receptor to activate the downstream signal transduction. In conclusion, the 3D printed Ti-5Cu alloy is a promising biomaterial to be applied to repair various bone defects for its good bone immunomodulatory properties, but we still need further *in vivo* experiments to confirm it.

## Data availability statement

The raw data supporting the conclusions of this article will be made available by the authors, without undue reservation.

## Author contributions

GZ and QW: supervision. XZa: methodology, writing—review, editing, original draft, and data analysis. XZo and DX: writing—graph and materials preparation. HS: data analysis. HXS and YS: writing—review and editing. All authors contributed to the article and approved the submitted version.

## Funding

This work was supported by the National Natural Science Foundation of China (51871050), Natural Science Foundation Project of Liaoning Province (2020-MS-150, 2018225059), and Shenyang Science and Technology Funded Project (RC190290).

## Conflict of interest

The authors declare that the research was conducted in the absence of any commercial or financial relationships that could be construed as a potential conflict of interest.

## Publisher’s note

All claims expressed in this article are solely those of the authors and do not necessarily represent those of their affiliated organizations, or those of the publisher, the editors and the reviewers. Any product that may be evaluated in this article, or claim that may be made by its manufacturer, is not guaranteed or endorsed by the publisher.
