# Supplementary figures and images for: 3D printed Ti-5Cu alloy accelerates osteogenic differentiation of MC3T3-E1 cells by stimulating the M2 phenotype polarization of macrophages
Source: Front Immunol. 2022 Oct 7;13:1001526. doi: 10.3389/fimmu.2022.1001526 (PMC9585254; doi:10.3389/fimmu.2022.1001526)

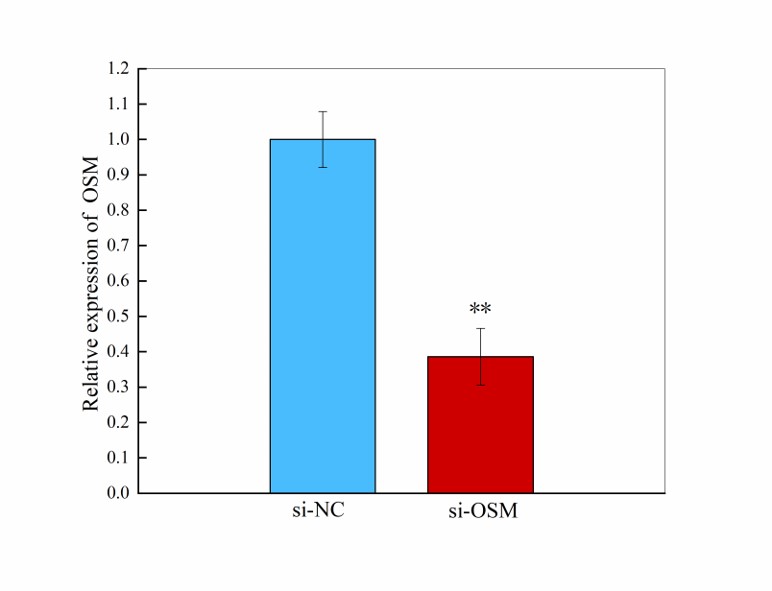


The gene expression of OSM in RAW264.7 cells measured by RT-qPCR. (n=3, ** p < 0.01)

Supplement: Supplementary file 1 [file DataSheet_1.docx]
